# HSI-AgriFoodAnomaly, a hyperspectral dataset for foreign object detection in agri-food inspection

**DOI:** 10.1038/s41597-026-07835-7

**Published:** 2026-07-15

**Authors:** Mohammed El Amine Bechar, Nadine Abdallah Saab, Olga Assainova, Hafsa El Hafyani, Nesma Settouti, Marwa Elbouz

**Affiliations:** 1 LabISEN, LSL, ISEN Ouest, 29200 Brest, France; 2 LabISEN, LSL, ISEN Ouest, Lebanese University, Lebanon; 3 LabISEN, LSL, ISEN Ouest, 14000 Caen, France

## Abstract

Ensuring food safety in agri-food production requires reliable inspection data for detecting foreign objects and other contamination risks. Hyperspectral imaging (HSI) is a relevant non-destructive modality for this purpose because it combines spatial information with detailed spectral responses. This Data Descriptor presents HSI-AgriFoodAnomaly, an open hyperspectral image dataset acquired under conveyor-based industrial-like conditions for foreign object annotation in an oat, white-chocolate and dark-chocolate mixture. The dataset contains 147 calibrated hyperspectral cubes, associated red-green-blue (RGB) renderings, pixel-level binary masks, and polygon annotations. Each hyperspectral cube was acquired in the visible to near-infrared range, with 300 contiguous spectral bands covering approximately 381–1016 nm. The annotated foreign objects (FOs) include textile and fibre-based materials, plastics, paper-based materials, metals, wood and plant residues, minerals, glass, mixed-object scenes, and anomaly-free scenes. The dataset is organised into training, validation and test subsets at the cube level, in order to prevent data leakage between splits. HSI-AgriFoodAnomaly can be reused for hyperspectral image analysis, foreign object localisation, binary classification, object detection, semantic segmentation, and benchmarking of data processing pipelines. The dataset and the accompanying code are publicly available.

## Background & Summary

Ensuring food safety and quality remains a global priority as agri-food industries face increasing demands for efficient, automated, and reliable inspection systems. *Hyperspectral imaging* (HSI) has emerged as a powerful non-invasive modality, combining the spatial detail of imaging with the spectral resolution of spectroscopy. Unlike conventional RGB imaging, HSI captures hundreds of contiguous spectral bands, which can provide information on material composition, surface properties, and spectral differences that are not directly observable from visible colour alone. HSI imaging, initially developed for remote sensing^1^, has expanded to fields such as precision agriculture^2^, environmental monitoring^3^, and more recently, food inspection^4^. In agri-food production, HSI enables the identification of anomalies, such as, surface defects, bruises, microbial contamination, and foreign objects (FOs) that often go undetected using traditional imaging methods^5–9^.

Recent advances in machine learning, particularly deep learning, have increased the interest in HSI-based inspection systems. However, progress is hindered by the lack of publicly available, well-annotated datasets specifically tailored to anomaly detection in agri-food contexts. Most existing datasets are often proprietary or restricted, focus on specific product categories or defect types, use simplified acquisition setups, or lack the variability and annotation detail needed for reproducible research. For Data Descriptor purposes, this background therefore focuses on existing HSI resources and application contexts that motivate the need for reusable, well-documented, pixel-level annotated hyperspectral data in agri-food inspection.

HSI has been investigated for foreign-object detection in several industrial food inspection settings. Medus *et al*.^10^ studied sealing-zone contamination in polyethylene terephthalate (PET)-tray packaging using an inline HSI system. Kwak *et al*.^11^ addressed foreign object detection in seaweed sheets using visible and near-infrared (VNIR) hyperspectral imaging, while Lohumi *et al*.^12^ combined multispectral fluorescence and RGB imaging for foreign-object detection in fresh-cut vegetables. Tunny *et al*.^13^ further contributed multimodal spectral data for foreign material detection in vegetables, including VNIR, short-wave infrared (SWIR), and fluorescence modalities. Shu *et al*.^14^ investigated foreign objects in chili peppers, including visually similar red contaminants, and Chen *et al*.^8^ used near-infrared (NIR) hyperspectral imaging for defect inspection in green coffee beans. More recently, Ghimpeteanu *et al*.^15^formulated foreign-object detection on pork belly as a semantic-segmentation problem using near-infrared hyperspectral imaging. Together, these studies illustrate the relevance of HSI for foreign object detection across diverse product matrices, contaminant types, and acquisition configurations.

Beyond foreign object detection, HSI has also been widely used for plant disease diagnosis and postharvest quality assessment, which are closely related to the broader problem of detecting subtle or non-visible anomalies in biological and food products. For plant disease diagnosis, HSI has been applied to early bacterial leaf blight detection in rice^16^, gray mold diagnosis in strawberry leaves^17^, asymptomatic potato late blight detection^9^, and corn disease classification^18^. For postharvest quality assessment, HSI-based approaches have been investigated for strawberry anomaly detection^19^, lemon bruise detection^20^, and spinach juice residue detection on stainless steel surfaces^21^. These works show that hyperspectral data can capture information related to defects, contamination, physiological stress, or material residues, but they also highlight the diversity of acquisition protocols, spectral ranges, target products, and annotation strategies used in the field.

Several hyperspectral datasets and studies have therefore contributed to the development of HSI-based analysis in agricultural and food-related contexts. Examples include cotton foreign matter classification^22^, soybean charcoal rot detection^23^, peanut defect classification^24^, blueberry mildew detection^25^, grapevine virus detection^26^, seed stress phenotyping^27^, polyethylene terephthalate (PET)-tray contamination inspection^10^, seaweed foreign-object detection^11^, vegetable foreign material detection^12,13^, coffee bean defect inspection^8^, strawberry anomaly detection^19^, lemon bruise detection^20^, potato late blight detection^9^, corn disease classification^18^, chili foreign object detection^14^, and pork belly foreign object segmentation^15^. A comparative overview of representative studies and datasets is provided in Table 1, with emphasis on application domain, spectral range, dataset size, defect or foreign object categories, and data characteristics.

**Table 1:** Representative hyperspectral imaging studies and datasets related to anomaly, defect, disease, and foreign object analysis in agricultural and food inspection contexts.

Application group	Representative studies	Typical data characteristics	Relation to HSI-AgriFoodAnomaly
Foreign object or foreign matter inspection	PET trays^10^, seaweed^11^, fresh-cut vegetables^12,13^, chili peppers^14^, pork belly^15^.	Food-specific HSI or multispectral data with heterogeneous foreign materials and acquisition settings.	Motivates reusable datasets with calibrated HSI cubes, aligned RGB images, pixel-level masks, and documented splits.
Product defects and postharvest quality	Peanuts^24^, coffee beans^8^, strawberries^19^, lemons^20^, blueberries^25^, spinach residues^21^.	Product-specific datasets focused on defects, bruises, decay, residues, or contamination.	Complements HSI-AgriFoodAnomaly, which focuses on foreign objects embedded in a heterogeneous food matrix.
Plant disease, stress, and phenotyping	Soybean^23^, rice^16^, strawberry leaves^17^, grapevine^26^, seeds^27^, potato^9^, corn^18^.	Spectra, patches, ROIs, sub-images, canopy images, or sample-level annotations.	Illustrates the broader value of HSI for subtle anomaly detection, but differs from industrial foreign object inspection.
Spectral-sample or patch-level resources	Cotton foreign matter spectra^22^, soybean patches^23^, potato sub-images^9^, chili pixel samples^14^, vegetable foreign-material spectra^13^.	Data often described as spectra, patches, ROIs, pixels, or object samples rather than complete annotated scenes.	HSI-AgriFoodAnomaly provides full HSI scenes with spatially aligned RGB renderings and pixel-level binary masks.
HSI-AgriFoodAnomaly	This scientific data^28^.	147 calibrated HSI cubes, RGB renderings, polygon annotations, binary masks, normal scenes, mixed-object scenes, and cube-level splits.	Designed for reusable foreign object annotation and detection studies in conveyor-based agri-food inspection.

Despite their value, many existing resources remain limited for reusable benchmarking in agri-food foreign object detection. First, several studies are focused on narrow product matrices or specific defect categories, such as cotton foreign matter^22^, soybean charcoal rot^23^, peanut defects^24^, blueberry mildew^25^, grapevine virus detection^26^, seed stress phenotyping^27^, coffee bean defects^8^, strawberry anomalies^19^, lemon bruises^20^, potato late blight^9^, corn leaf diseases^18^, or spinach juice residues^21^. Second, some resources are primarily provided or described as spectral samples, patches, sub-images, droplets, beans, or region-level data rather than full hyperspectral scenes with pixel-level masks, as in cotton spectra^22^, soybean patches^23^, peanut kernel images^24^, grapevine canopy images^26^, strawberry leaf regions of interest (ROIs)^17^, potato sub-images^9^, vegetable foreign-material spectra^13^, and coffee bean-level inspection data^8^. Third, for foreign object inspection specifically, available studies cover valuable but heterogeneous acquisition settings, including PET trays^10^, seaweed sheets^11^, fresh-cut vegetables^12,13^, chili peppers^14^, and pork belly^15^, with differences in spectral range, imaging modality, annotation granularity, and dataset structure. In addition, acquisition conditions, file formats, metadata, annotation protocols, and split definitions are not always documented in a standardised way across these resources, which can make independent reuse and benchmarking more difficult. These limitations motivate the creation of open hyperspectral datasets that combine calibrated HSI cubes, aligned RGB renderings, pixel-level annotations, clear file organisation, and documented quality-control procedures.

This Data Descriptor presents *HSI-AgriFoodAnomaly*, an open hyperspectral image dataset designed to support research on foreign object annotation and detection in conveyor-based agri-food inspection. The dataset focuses on a representative inspection case involving an oat-based food matrix composed of oat flakes, white chocolate chips, and dark chocolate chips. This matrix was selected because it introduces heterogeneous texture, colour, reflectance, and spatial clutter, which are relevant to foreign object localisation in realistic inspection scenarios. The dataset contains 147 calibrated hyperspectral cubes acquired under industrial-like line-scan conditions using a pushbroom VNIR hyperspectral camera. It includes foreign-object-free scenes and scenes containing foreign objects from several material families, including textile and fibre-based materials, plastics, paper-based materials, metals, wood and plant residues, minerals, glass, and mixed-object configurations.

For each hyperspectral cube, the dataset provides calibrated HSI data, an RGB rendering, pixel-level binary masks, and polygon annotations. The data are organised into training, validation, and test subsets at the cube level. The dataset can be reused for hyperspectral image analysis, foreign object localisation, binary classification, object detection, semantic segmentation, and evaluation of preprocessing or modelling pipelines. By documenting the acquisition protocol, annotation strategy, file organisation, metadata, and technical validation procedures, this Data Descriptor aims to facilitate the reuse of *HSI-AgriFoodAnomaly* for hyperspectral image analysis and agri-food inspection research.

Table 1 summarises representative HSI studies and datasets from a data-reuse perspective, including application domain, data characteristics, and annotation granularity. Building on these observations, *HSI-AgriFoodAnomaly* provides a documented resource for foreign object analysis in agri-food inspection. Its main reusable components are:

- 147 calibrated hyperspectral cubes acquired under conveyor-based industrial-like conditions;
- corresponding RGB renderings provided for visual inspection and conventional image-based workflows;
- pixel-level binary masks and polygon annotations for foreign object localisation;
- foreign objects grouped into material-based categories, including textile/fibre, plastics, paper, metals, wood/plant residues, minerals/glass, and mixed-object scenes;
- anomaly-free scenes that can be used as negative controls;
- a cube-level train, validation, and test split, ensuring strict data isolation across subsets.

## Methods

### Acquisition Equipment and Setup

All hyperspectral acquisitions were performed under line-scan conditions using a professional pushbroom hyperspectral system to emulate industrial inspection settings, within a controlled and light-isolated environment to ensure consistency and repeatability. The setup comprised:

- **Camera:***A Resonon Pika L* hyperspectral camera operating in the visible to near-infrared range, from 381nm to 1016nm, capturing 300 contiguous spectral bands with a spectral resolution of 2.1nm. RGB images can be derived from the acquired HSI cubes using SpectrononPro’s built-in spectral-to-RGB rendering function, which integrates spectral information across the visible range (381–700 nm). No visual artefacts were observed in the generated RGB images, which present natural colour rendering throughout the dataset. RGB outputs are directly provided in the dataset in PNG and TIFF formats.
- **Illumination:** Four halogen light sources, positioned obliquely around the scene, provided diffuse and uniform lighting while minimizing specular reflections.
- **Conveyor system:** A motorized conveyor belt synchronized with the camera’s frame rate enabled line-by-line scanning along the Y-axis, replicating the motion of real conveyor-based inspection systems.
- **Resolution and field of view:** The camera provided a spatial resolution of 900 pixels for each scanned line (X-axis). Each HSI cube covered approximately 12.35cm along the Y-axis and 11.11cm along the X-axis, with a spatial resolution of 1000 × 900 pixels, corresponding to an approximate spatial sampling of 0.123 mm per pixel along both spatial axes.

**Fig. 1:**
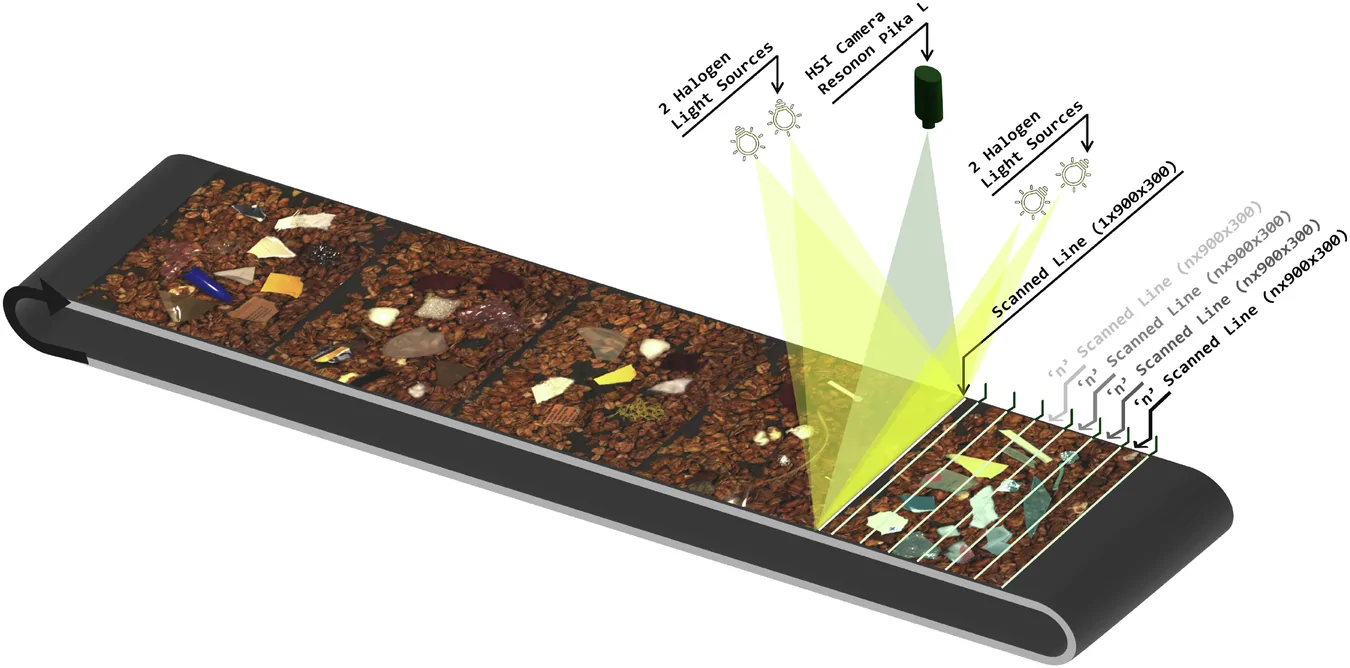
Acquisition setup: A pushbroom hyperspectral camera captures the conveyor line-by-line.

The entire acquisition setup (as illustrated in Fig. 1) was enclosed in a light-isolated chamber, eliminating ambient light variability and ensuring reproducible illumination conditions across all acquisition sessions.

### Radiometric Calibration

Prior to each acquisition session, reflectance calibration was performed using SpectrononPro (version 3.4.6, Resonon Inc., Bozeman MT, USA). The lamp was first powered on and allowed to stabilise for a minimum of 20 minutes. A dark current correction was then recorded by placing the lens cap on the camera. A white reference was subsequently acquired using a Spectralon tile supplied by the manufacturer, placed perpendicularly on the conveyor platform. Calibrated reflectance was computed automatically according to:

1 $$\,{\rm{Reflectance}}=\frac{{\rm{Raw\; Data}}-{\rm{Dark\; Correction}}}{{\rm{White\; Reference}}-{\rm{Dark\; Correction}}}$$

where *Raw Data* is the sensor signal, *Dark Correction* is the response with no illumination, and *White Reference* is the Spectralon tile response. Calibrated values are nominally in [0, 1]; values slightly exceeding 1.0 may occur near sensor saturation, which is standard behaviour in push-broom HSI systems.

### Foreign Object Categories

The food background used throughout the dataset consisted of a heterogeneous mixture of oat flakes, white chocolate chips, and dark chocolate chips. This composition was selected for its high intra-scene variability in texture, colour, reflectance, and structure, factors that increase the detection challenge and closely mimic the visual complexity of real agri-food production lines. Scenes were acquired with varying product densities, ranging from sparse to dense layouts, to simulate the natural flow variability encountered on conveyor belts. Foreign objects were selected to represent non-food contaminants commonly encountered on agri-food production lines. These FOs were categorized into six material-based classes, each chosen for their spectral diversity, industrial plausibility, and visual ambiguity relative to food products:

1. **Textile and fiber-based materials:** Includes fabric pieces, medical cotton, netting (3 types), and textile threads (3 types). These soft, fibrous materials often exhibit irregular textures and tend to blend visually with food components.
2. **Plastics:** Includes pen casing fragments and six different plastic films, varying in color, transparency, thickness and flexibility. These simulate contamination scenarios from packaging residues or machine parts.
3. **Paper-based materials:** Comprises six types of paper such as kraft, printed, and coated paper, representing labeling debris or packaging fragments frequently found in industrial environments.
4. **Metals:** Consists of aluminum foil, metal nails, paper clips, and metallic sponge fragments. These represent high-risk contaminants typically introduced by equipment failure, maintenance, or mechanical wear.
5. **Wood and plant-based materials:** Covers small skewer fragments and dried plant stems, which are relevant in contexts involving raw agricultural input handling or biodegradable packaging materials.
6. **Minerals and glass:** Includes small stones and glass shards, high-risk anomalies typically resulting from transportation, storage, or ingredient processing stages.

This diversity in material, geometry, and scene context was designed to provide a realistic testbed for evaluating hyperspectral models under challenging conditions. Table 2 provides a summary of the six FO categories with representative RGB image samples.

**Table 2:** Overview of foreign-object categories with representative RGB image samples.

### Controlled Scene Generation and Acquisition Protocol

Each acquisition scene was carefully composed to simulate industrial production environments. Each FO category was recorded in multiple configurations to increase intra-class variability, including variations in object count, spatial positioning (center, periphery), and visibility (fully visible, partially occluded, or embedded within the food matrix). Certain FO types (e.g., transparent plastics, textile threads, glass shards) were deliberately chosen due to their visual similarity to common food textures, thereby increasing the detection complexity. The following scene parameters were systematically varied:

- **Product base density:** The oat–chocolate mixture serving as the background was adjusted from sparse to dense layouts, simulating product flow variability typically encountered on conveyor belts.
- **Single vs. multiple FOs:** Scenes were composed with either a single FO type or a combination of FOs from different categories, increasing the semantic and spectral complexity of the detection task.
- **FO positioning and occlusion:** FOs were placed at various locations across the scene (center, edge, or overlapping with dense product clusters), with different levels of visibility: fully exposed, partially occluded, or deeply embedded, simulating the partial occlusions and spatial randomness typical in real production lines.

A set of foreign-object-free scenes (referred to as “normal” scenes) was also included, reflecting uncontaminated production states and serving as negative controls during model training and evaluation.

### Pixel-Level Annotation Protocol

To enable supervised learning across a wide range of computer vision tasks, a manual, high-precision annotation protocol was implemented, focusing on FO localisation at the pixel level. All annotations were performed entirely manually using the open-source tool *Label Studio* (https://github.com/HumanSignal/label-studio), chosen for its flexibility, support for custom formats, and open-source reproducibility.

Each HSI cube was first rendered as an RGB image using SpectrononPro (see the Acquisition Equipment and Setup subsection) to provide a visual reference for the annotator. For foreign objects exhibiting low contrast in the RGB rendering (e.g., transparent plastic films, thin textile threads, or glass shards), additional spectral bands were examined to identify wavelength ranges where the FO signature was most discriminative against the food background. This multi-band visual inspection ensured accurate delineation of ambiguous or partially transparent objects.

Each FO was precisely delineated using polygon-based annotations in Label Studio, which were subsequently converted to pixel-level binary masks. Each mask pixel carries one of two values: 255 (foreground), indicating the presence of a foreign object, or 0 (background), indicating the food matrix or the absence of any FO. Importantly, all foreign objects are assigned the same foreground value regardless of their material category, resulting in a binary annotation scheme rather than a multi-class labelling.

All annotations were performed by a single trained annotator. Upon completion, the full set of annotations was independently reviewed by a second annotator, who verified the accuracy of object boundaries, the consistency of labelling across scenes, and the correct handling of edge cases such as partial occlusions and spectrally ambiguous objects. Any annotation flagged during this review was corrected before inclusion in the final dataset. During the second-annotator review, approximately 5 to 6 scenes out of 147 required minor correction, corresponding to less than 4% of the dataset. These corrections mainly concerned boundary refinement for spectrally ambiguous materials, such as transparent plastics and thin textile threads. No annotation was fully rejected or redrawn. Since no formal inter-annotator agreement metric was computed during the original annotation process, this correction rate should be interpreted as an approximate indication of annotation stability rather than as a formal agreement score.

The annotation protocol supported multiple downstream tasks:

- **Binary classification:** Presence or absence of FOs per patch or scene.
- **Object detection:** Bounding boxes can be derived from the JSON polygon annotations by computing the minimum and maximum coordinates of each polygon vertex, i.e., ${x}_{\min }=\min ({x}_{i})$, ${x}_{\max }=\max ({x}_{i})$, ${y}_{\min }=\min ({y}_{i})$, and ${y}_{\max }=\max ({y}_{i})$.
- **Semantic segmentation:** Pixel-wise annotations support fine-grained FO segmentation.

To ensure full scene coverage, all scenes, including foreign-object-free ones, were annotated with blank masks (all-zero), enabling models to learn both normal and FO-containing patterns. All masks are spatially aligned with their corresponding RGB images and HSI cubes, facilitating pixel-wise learning across modalities.

Examples of annotated images, binary masks, and overlay visualisations are shown in Table 3, illustrating the wide range of spatial distributions, FO sizes, occlusion levels, and object shapes captured in the dataset.

**Table 3:** Example of annotated scenes: original RGB image, corresponding binary mask, and overlay of the mask on the RGB image.

## Data Records

The HSI-AgriFoodAnomaly dataset is publicly available at the French national research data repository *Recherche Data Gouv* under the persistent identifier https://doi.org/10.57745/QTLG7X^28^. The dataset is released under a Creative Commons Attribution 4.0 International (CC BY 4.0) licence.

### File Formats and Folder Structure

The dataset is partitioned into three top-level splits (train, val, test), each sharing an identical internal structure composed of three sub-directories:

- HSI-Hypercube/ calibrated hyperspectral image cubes stored in Band Interleaved by Line (BIL) format. Each scene is represented by a pair of files: a .bil binary file containing the raw spectral data, and a .bil.hdr header file encoding acquisition metadata.
- Annotation/ pixel-level binary segmentation masks, available in two formats:
  – PNG/ one 8-bit grayscale .png mask per scene.
  – JSON/ one .json file per use case, containing the raw polygon annotations as exported from Label Studio.
- RGB/ RGB images derived from the HSI cubes via SpectrononPro’s built-in spectral-to-RGB rendering function, provided in two formats:
  – PNG/ one 8-bit, 3-channel .png file per scene.
  – TIFF/ one 16-bit, 3-channel .tiff file per scene, preserving higher radiometric precision.

The complete folder structure is as follows:



### HSI Cube Files

Each cube has the following dimensions: 1000 lines (Y-axis, scan direction)  × 900 samples (X-axis, spatial)  × 300 bands (spectral). Data values are stored as 16-bit unsigned integers (data type = 12) with a reflectance scale factor = 10000, meaning that physical reflectance values are obtained by dividing the stored integers by 10, 000.

The header file specifies the full list of 300 calibrated wavelengths, covering the range from 381nm to 1016nm (VNIR range), with a mean spectral sampling interval of approximately 2.1nm. Key metadata fields are summarised in Table 4.

**Table 4:** Key metadata fields from the ENVI header files (.bil.hdr).

Field	Value
interleave	bil (Band Interleaved by Line)
data type	12 (16-bit unsigned integer)
lines	1000
samples	900
bands	300
byte order	0 (little-endian)
wavelength units	nm
wavelength range	381 – 1016 nm
reflectance scale factor	10000
framerate	17.80 frames per second (fps)
imager serial number	100121-159 (Resonon Pika L)

### Annotation Files

Pixel-level annotations are provided in two complementary formats:

**PNG masks**. Each .png mask is an 8-bit single-channel grayscale image of size 1000 × 900 pixels, spatially aligned with its corresponding HSI cube and RGB image. Pixel values follow a binary encoding scheme:

- **0** – background, corresponding to the food matrix or to an empty scene with no FO present;
- **255** – foreground, corresponding to the presence of any foreign object, regardless of material category.

Masks are therefore binary and category-agnostic: all FO categories share the same foreground value (255). Category information is not encoded in the mask pixel values but is instead recoverable from the scene filename and folder structure. Anomaly-free, or normal, scenes are provided with all-zero masks.

**JSON annotations**. Each .json file contains the raw polygon annotations exported from Label Studio for the corresponding split subset. Each annotation entry encodes the polygon vertices in percentage coordinates relative to the image dimensions, the annotated label ("FO"), and the scene filename. These JSON files can be used to regenerate masks at any desired resolution or to access the original polygon boundaries directly.

For object-detection tasks, axis-aligned bounding boxes can be derived deterministically from the JSON polygon annotations. Since Label Studio stores polygon vertices as percentage coordinates, each vertex (*x*_*i*_, *y*_*i*_) is first converted to pixel coordinates using the image width *W* = 900 and height *H* = 1000:

$${x}_{i}^{{\rm{px}}}=\frac{{x}_{i}}{100}\times W,\qquad {y}_{i}^{{\rm{px}}}=\frac{{y}_{i}}{100}\times H.$$

The bounding box associated with a polygon is then obtained by computing the minimum and maximum pixel coordinates of its vertices:

$${x}_{\min }=\mathop{\min }\limits_{i}\left({x}_{i}^{{\rm{px}}}\right),\quad {x}_{\max }=\mathop{\max }\limits_{i}\left({x}_{i}^{{\rm{px}}}\right),\quad {y}_{\min }=\mathop{\min }\limits_{i}\left({y}_{i}^{{\rm{px}}}\right),\quad {y}_{\max }=\mathop{\max }\limits_{i}\left({y}_{i}^{{\rm{px}}}\right).$$

The resulting rectangle defines the top-left and bottom-right corners of the object-level bounding box. These derived bounding boxes can be used for standard object-detection workflows, while the original polygons and binary masks remain available for more precise segmentation tasks.

### RGB Image Files

For each HSI cube, a corresponding RGB image is provided in both PNG (8-bit) and TIFF (16-bit) formats, spatially aligned with the hyperspectral data and annotation masks. These images were generated using SpectrononPro’s built-in spectral-to-RGB rendering function (see the Acquisition Equipment and Setup subsection) and are intended to facilitate visual inspection, qualitative evaluation, and use by researchers without specialised HSI processing tools.

### Data Overview

The dataset comprises 147 calibrated HSI cubes, split at the cube level into three subsets: 89 for training, 17 for validation, and 41 for testing. This partitioning strategy ensures no spatial or spectral overlap between splits, thereby preventing data leakage.

Table 5 provides the distribution of cubes across data subsets, covering single-object scenes, multi-object scenes, and anomaly-free configurations. The test set additionally includes one scene featuring previously unseen FO types not present in the training or validation subsets, to support evaluation of model generalisation to novel contaminants. It is important to note that although Table 5 organises scenes by material family for descriptive purposes, the dataset does not provide multi-class annotations. All FO instances are annotated with a single binary label: pixels belonging to any foreign object are marked as foreground (value 255), regardless of material type, while all remaining pixels are marked as background (value 0).

**Table 5:** Distribution of foreign-object and anomaly-free samples across training, validation, and test sets.

FO Category	Train	Val	Test	Total
Textile and fiber-based materials	40	8	16	64
Plastics	20	4	8	32
Paper-based materials	5	1	2	8
Metals	5	1	2	8
Wood and plant-based materials + Minerals and glass	5	1	2	8
Mixed (multi-object scenes)	5	1	7	13
Normal (anomaly-free)	9	1	3	13
New foreign-object types	—	—	1	1
**Total**	**89**	**17**	**41**	**147**

**Fig. 2:**
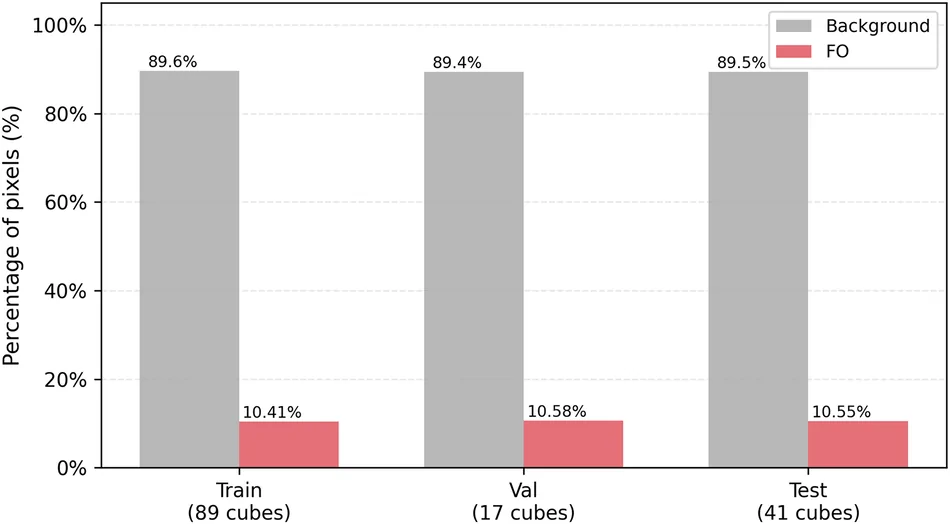
Pixel-level class distribution across dataset splits. FO pixels account for approximately 10.5% of all pixels per split, with the remaining pixels corresponding to the food background.

At the pixel level, foreign object annotations represent approximately 10.5% of all pixels across the three subsets, with the remaining 89.5% corresponding to the food background, as illustrated in Fig. 2. This class imbalance is consistent across splits and reflects the realistic proportion of FO contamination encountered in industrial inspection scenarios. Users should note that some material categories contain a limited number of scenes (5–8 cubes). Category-level statistical inferences from these categories should therefore be interpreted with caution, and per-category performance conclusions should not be drawn from the baseline experiment presented in the Technical Validation section.

## Technical Validation

The technical validation of the *HSI-AgriFoodAnomaly* dataset is structured around two complementary assessments: a spectral quality analysis confirming the discriminative richness of the hyperspectral data, and a usability baseline experiment comparing the detection performance of artificial intelligence (AI) models trained on HSI cubes versus RGB images.

### Spectral Quality Assessment

**Fig. 3:**
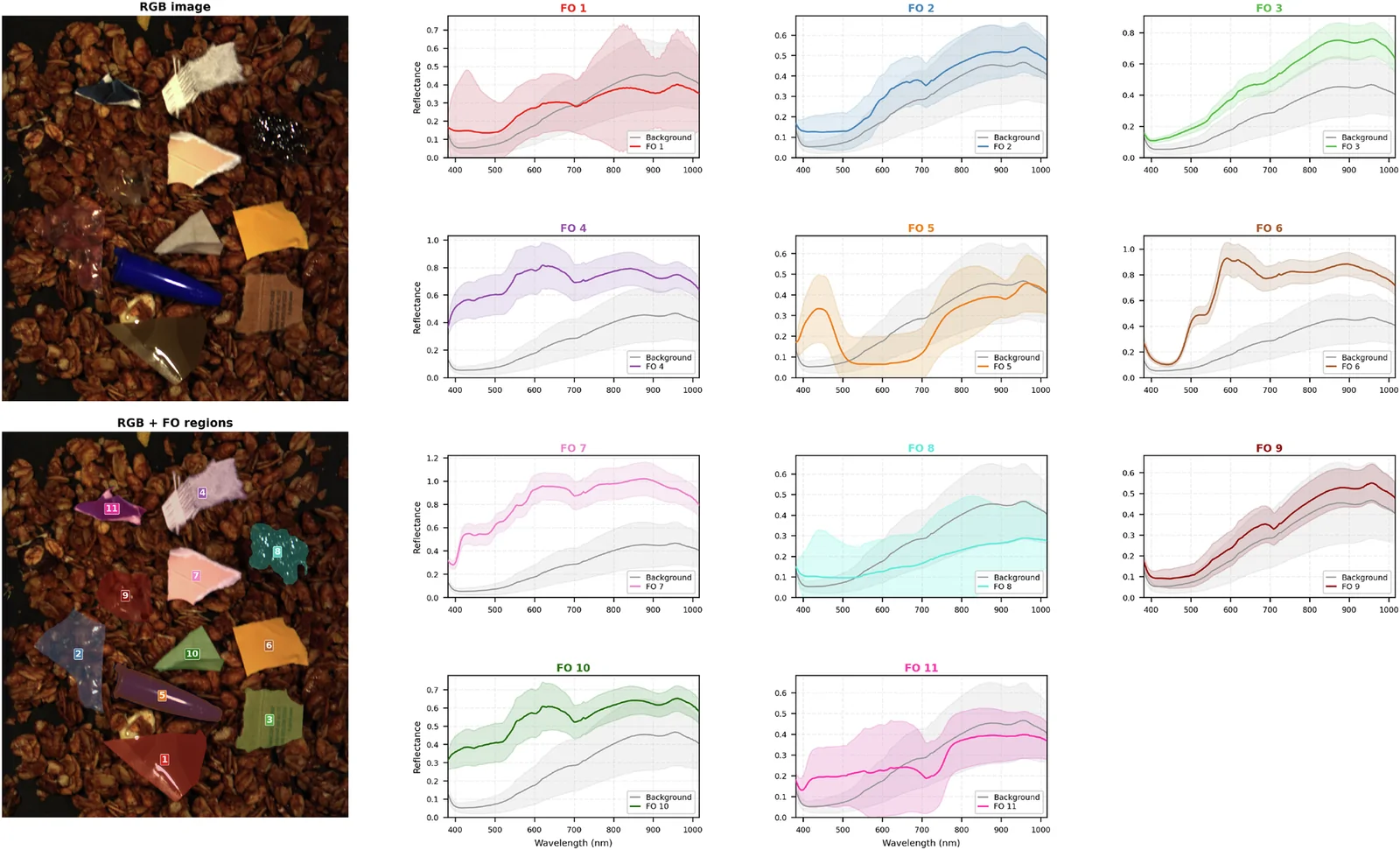
Mean spectral signatures per FO region versus food background for *LM_1*. Left panel: RGB image; centre panel: RGB image with FO regions coloured and numbered; right panels: mean reflectance  ± std per region over the full spectral range (381–1016 nm).

**Fig. 4:**
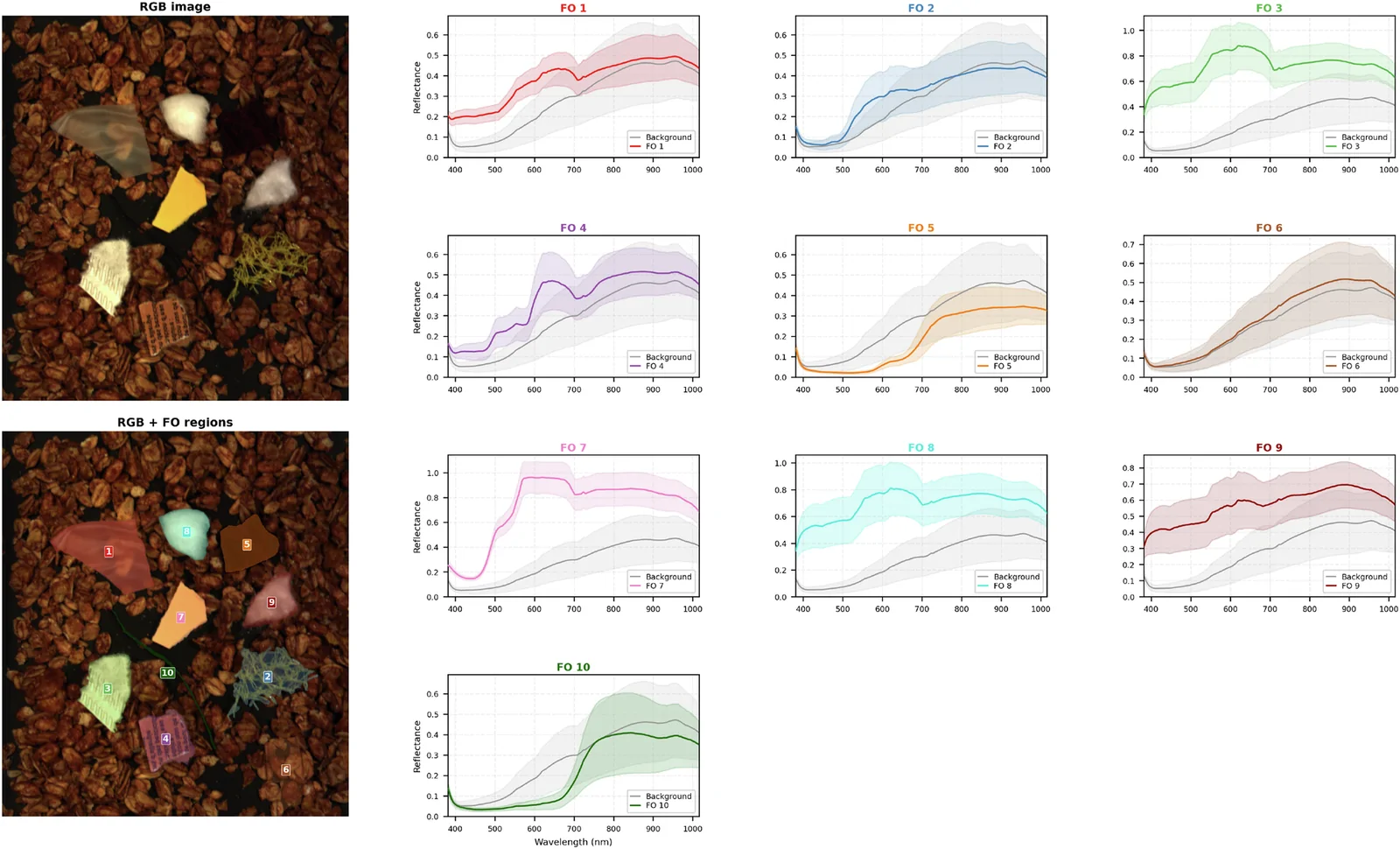
Mean spectral signatures per FO region versus food background for *LM_2*. Left panel: RGB image; centre panel: RGB image with FO regions coloured and numbered; right panels: mean reflectance  ± std per region over the full spectral range (381–1016 nm).

To assess the spectral discriminability of the dataset, mean spectral signatures were extracted for each annotated FO region and compared against the food background signature across two representative mixed-object scenes (*LM_1* and *LM_2*). For each scene, all FO regions were identified from the binary annotation masks and their mean reflectance  ± standard deviation was computed over the full spectral range (381–1016 nm). The results are illustrated in Figs. 3 and 4.

The analysis indicates that the annotated FO regions considered in these two representative cubes exhibit spectral profiles that differ from the food background, supporting the spectral relevance of the dataset for FO analysis. However, the spectral contrast between FOs and the food background varies considerably depending on the FO category and the spectral range considered.

Several FO regions show strong contrast throughout the full spectral range, making them detectable by both RGB and hyperspectral imaging. In contrast, a subset of materials exhibit limited or near-zero contrast in the visible range (381–700 nm) yet become more distinguishable from the food background in the near-infrared range (NIR, 700–1016 nm), a spectral region inaccessible to conventional RGB cameras. In cube *LM_1*, 4 out of 11 FO regions (36%) fall into this category, with peak discriminability observed beyond 800 nm. Similarly, in cube *LM_2*, 2 out of 10 FO regions (20%) are detectable primarily in the NIR range, including one region whose reflectance is virtually indistinguishable from the background in the visible range yet presents a measurable spectral difference in the NIR.

To further quantify this separability, a band-wise separability index was computed for each of the 300 spectral bands across all three dataset splits, yielding 300 separability values per split. The index at band *b* is defined as:

2 $$\,{\rm{Sep}}\,(b)=\frac{| {\mu }_{{\rm{FO}}}(b)-{\mu }_{{\rm{BG}}}(b)| }{0.5\,({\sigma }_{{\rm{FO}}}(b)+{\sigma }_{{\rm{BG}}}(b))}$$

where *μ* and *σ* denote the mean and standard deviation of the reflectance values for FO and food background pixels at band *b*, respectively. Values of Sep ≥1.0 indicate good spectral separability at that band. Table 6 summarises key statistics derived from these 300 per-band values for each split: the maximum separability (${{\rm{Sep}}}_{\max }$), the mean separability across all 300 bands (Sep_mean_), the mean separability restricted to the visible range (Sep_VIS_, 381–700 nm), the mean separability restricted to the NIR range (Sep_NIR_, 700–1016 nm), and the number of bands achieving Sep ≥ 1.0. Results are consistent across splits.

**Table 6:** Summary statistics of the band-wise spectral separability index across dataset splits.

Split	${{\rm{Sep}}}_{\max }$	Sep_mean_	Sep_VIS_	Sep_NIR_	Bands Sep ≥ 1.0
Train	1.060	0.607	0.863	0.333	79/300
Val	1.259	0.728	0.989	0.448	82/300
Test	1.184	0.628	0.920	0.316	80/300

Sep_VIS_: mean separability over the visible range (381–700 nm); Sep_NIR_: mean separability over the NIR range (700–1016 nm).

These results reveal two complementary patterns. The visible range (381–700 nm) exhibits higher mean separability across all splits (Sep_VIS_ ≈ 0.86–0.99), indicating that many FO materials present distinct reflectance signatures from the food background in the spectral range accessible to RGB cameras. The NIR range (700–1016 nm) provides additional discriminative information not captured by the visible range alone: several FO regions that show limited contrast in the visible range exhibit clearer spectral differences beyond 700 nm, as illustrated by the representative spectral signatures in Figs. 3 and 4. This complementarity demonstrates that no single spectral sub-range is universally sufficient to discriminate all FO categories, and supports the use of the full 300-band spectral resolution of the dataset.

This observation highlights a critical limitation of RGB-based inspection systems: materials that appear visually similar to the food matrix under white light illumination may still carry distinct spectral signatures in wavelength ranges beyond human vision. The availability of 300 contiguous spectral bands in the *HSI-AgriFoodAnomaly* dataset therefore supports future studies on spectral feature selection, dimensionality reduction, and foreign-object detection beyond conventional RGB imaging.

### Dataset Usability Baseline

The experiments reported in this section are intended as dataset usability checks and should not be interpreted as a comprehensive benchmark of hyperspectral image classification, segmentation, band-selection, or real-time deployment methods. Although the *HSI-AgriFoodAnomaly* dataset supports multiple computer vision tasks, including object detection and semantic segmentation, the usability validation presented here focuses on patch-wise binary classification, which constitutes a fundamental and computationally tractable task for assessing whether the dataset can be exploited for deep learning. The objective is not to benchmark state-of-the-art architectures, but to provide a basic usability check showing that the dataset can be exploited for supervised foreign-object detection, while illustrating the potential added value of the hyperspectral modality compared with RGB-only inputs.

**Fig. 5:**
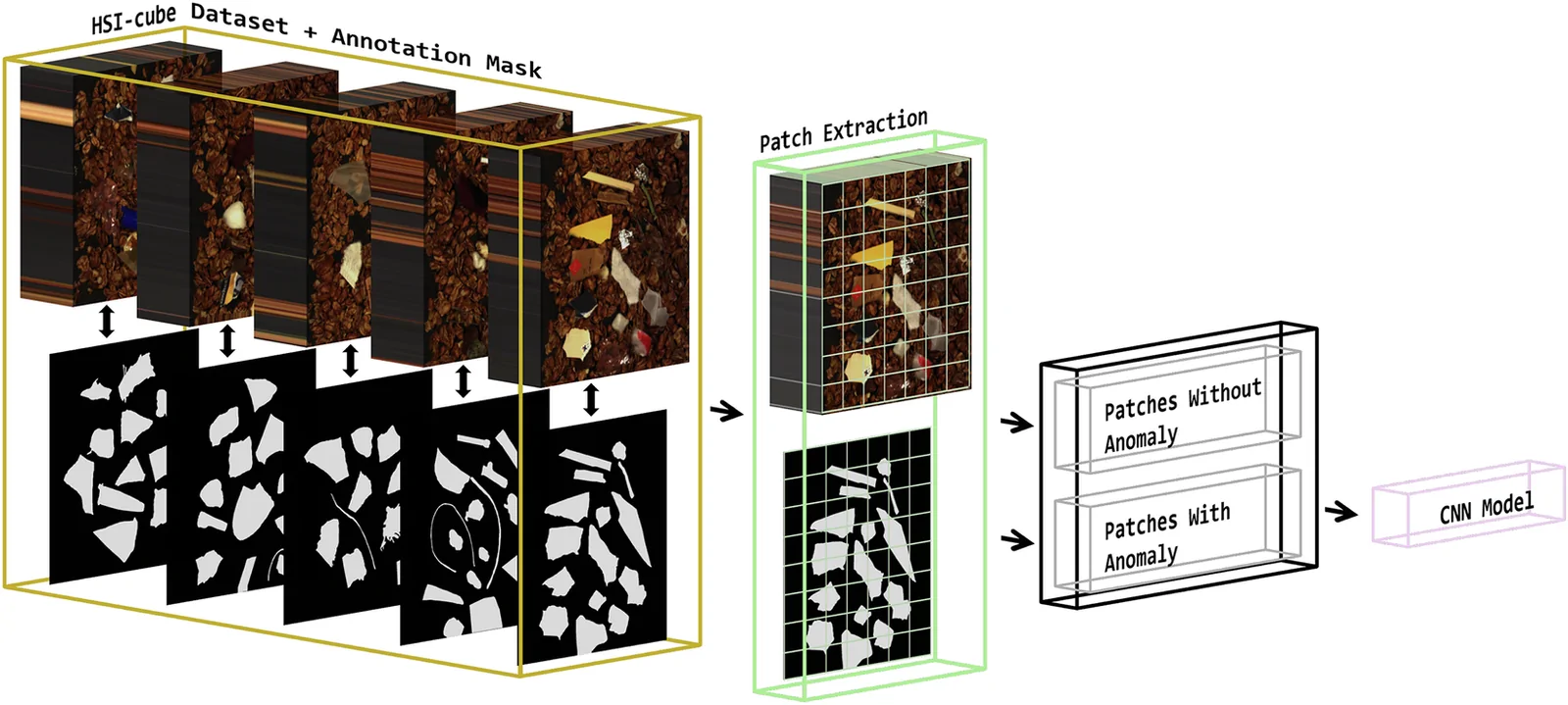
Validation pipeline: Labeled HSI cubes are patched and separated into patches with and without foreign objects. These labeled patches are used to train a CNN model for binary classification.

As illustrated in Fig. 5, the validation framework operates on a patch-wise basis: HSI cubes and their corresponding RGB images are partitioned into fixed-size spatial patches, each labelled as containing a foreign object or not based on the associated binary annotation mask. These labeled patches are then used to train a binary convolutional neural network (CNN) classifier to distinguish between FO-containing and FO-free patches. This patch-based approach reflects a conveyor-based inspection scenario in which sequential HSI lines are accumulated and analysed as spatial regions.

#### Patch Extraction

Patches of size 100 × 100 pixels were extracted from the full HSI cubes and their corresponding RGB images using a sliding window strategy with a stride of 50 pixels. Each patch was labelled as containing a foreign object or not, based on its associated binary annotation mask. The patch distribution across splits is reported in Table 7.

**Table 7:** Number of extracted patches at patch size 100 × 100 pixels per dataset split.

Split	With FO	Without FO	Total
Train	10,153	18,594	28,747
Val	2,171	3,320	5,491
Test	4,957	8,286	13,243

#### Model Architectures and Training Configuration

Two ResNet18 variants were evaluated and compared under identical training conditions. The **ResNet18-2D** was trained on RGB image patches using the timm library (https://github.com/rwightman/pytorch-image-models), accepting inputs of shape 3 × 100 × 100 and initialised with ImageNet-pretrained weights, as this architecture was originally designed for 3-channel RGB inputs. The **ResNet18-3D** was trained on full HSI patches using the MONAI library (https://monai.io), accepting inputs of shape 1 × 300 × 100 × 100, where the single input channel encodes the hyperspectral cube as a 3D volume with spectral bands serving as the depth dimension. This architecture explicitly captures spectral-spatial correlations through volumetric convolutions applied jointly across spatial and spectral dimensions, whereas the RGB-based 2D approach operates only on three integrated colour channels and therefore cannot exploit the full spectral depth of the HSI cube. Both models were trained under the same conditions, detailed in Table 8.

**Table 8:** Training configuration shared by both models.

Hyperparameter	Value
Device	NVIDIA RTX 6000 Ada Generation (48 GB)
Optimizer	Adam (*β*_1_ = 0.9, *β*_2_ = 0.999)
Loss function	BCEWithLogitsLoss
Epochs	100
Batch size	64
Learning rate	1 × 10^−4^ (fixed)
Data augmentation	None

#### Evaluation Metrics

To comprehensively evaluate model performance on the binary classification task (FO present vs. absent), the following metrics were computed on the test set:

- **Accuracy:** Overall proportion of correctly classified patches.
- **F1-score:** Harmonic mean of precision and recall, providing a balanced measure under class imbalance. The F1-score is computed for the positive (minority) class only, where the positive class corresponds to patches containing at least one foreign object.
- **AUC:** Area under the ROC curve, evaluating class separability across decision thresholds.
- **MCC:** Matthews Correlation Coefficient, a robust metric for imbalanced binary classification accounting for all four confusion matrix entries. As a scalar metric derived from all four entries of the confusion matrix, the MCC does not require class averaging and is not dominated by the majority class.

All metrics were computed on the test set using sigmoid-activated predictions at a decision threshold of 0.5.

#### Baseline Usability Results

Table 9 reports the test-set performance of both models. In this baseline experiment, the ResNet18-3D trained on full HSI data achieved higher test-set values than its RGB-based counterpart across the four reported metrics, with differences of +2.1% in accuracy, +2.0 points in F1-score, +2.0 points in AUC, and +4.0 points in MCC. These results suggest that the hyperspectral modality can provide additional discriminative information compared with RGB imaging in this experimental setting. The 3D architecture is designed to exploit spectral–spatial interactions in HSI data, whereas the RGB-based 2D approach operates only on three integrated colour channels.

**Table 9:** Comparative test-set performance of ResNet18-2D (RGB) and ResNet18-3D (HSI) at patch size 100 × 100 pixels.

Model	Input	Accuracy	F1-score	AUC	MCC
ResNet18-2D	RGB	0.903	0.872	0.940	0.798
ResNet18-3D	HSI (300 bands)	**0.924**	**0.892**	**0.960**	**0.838**

These results are presented as a usability baseline to confirm that the dataset contains sufficient discriminative information to train effective FO detection models. More comprehensive benchmarks using advanced HSI-specific architectures are left to future work.

**Fig. 6:**
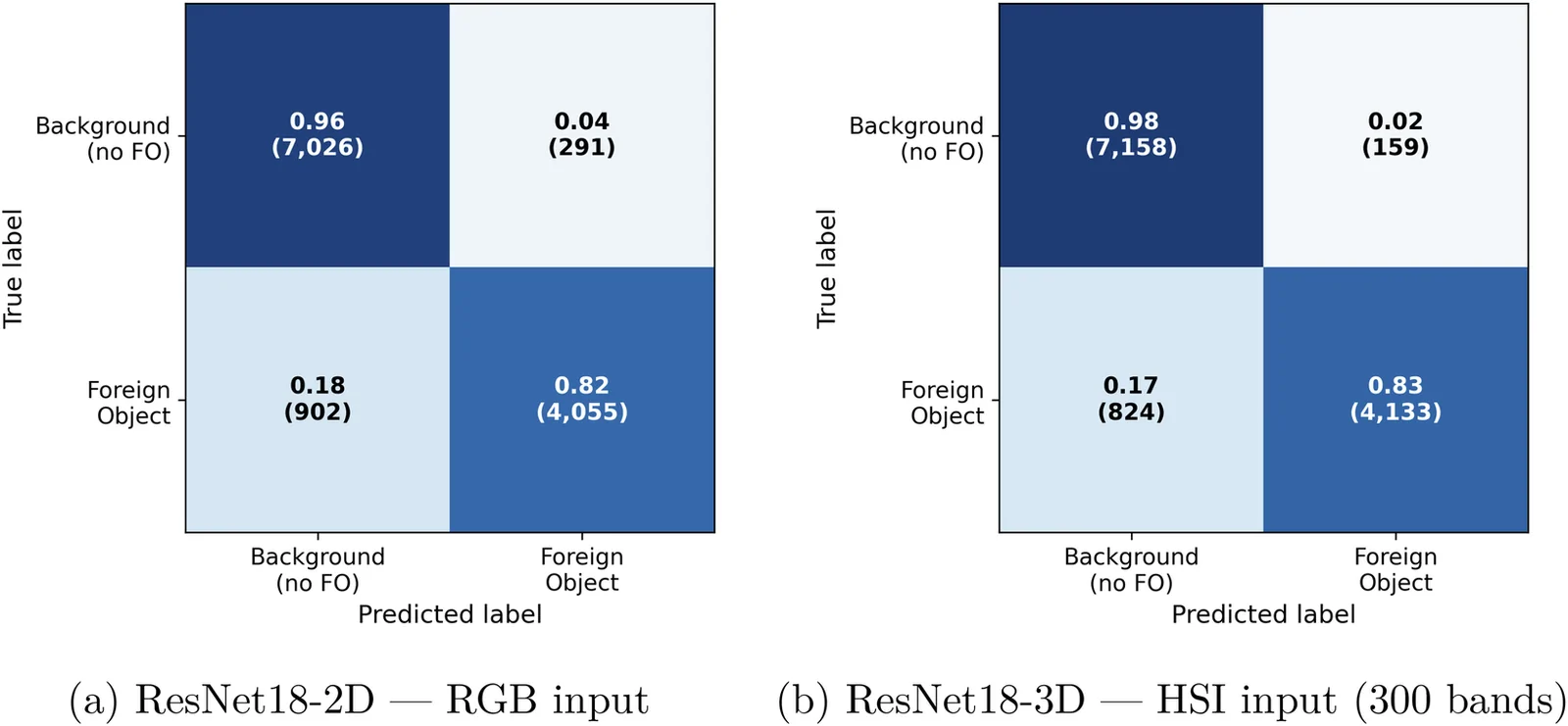
Confusion matrices on the test set for both baseline models. Values are row-normalised (recall-based); absolute patch counts are given in parentheses. Patch size: 100 × 100 pixels.

The confusion matrices for both models on the test set are shown in Fig. 6. Each matrix is row-normalised, with absolute patch counts given in parentheses. In this baseline experiment, the ResNet18-3D achieved a higher true positive rate and a lower false negative rate than the ResNet18-2D, suggesting that hyperspectral information may improve foreign-object detection sensitivity in this dataset. This observation is relevant for food safety applications, where missed detections may have higher practical consequences than false alarms.

To further support these quantitative results, qualitative analyses were conducted on selected test scenes (Table 10). In Example #3, which involves a small, partially occluded foreign object, the ResNet18-3D localises the foreign object in this illustrative example, whereas the ResNet18-2D fails to detect it, highlighting the sensitivity of hyperspectral data to subtle spectral variations that are not captured by RGB imaging alone. In Example #4, which features a previously unseen FO type not present during training, the ResNet18-3D detected the foreign object in this illustrative case, whereas the ResNet18-2D did not. These illustrative observations suggest that the full spectral information provided by the dataset may support more discriminative feature representations, particularly in challenging cases involving ambiguous materials, partial occlusions, or previously unseen foreign-object types.

**Table 10:** Foreign-object detection results on HSI cubes and RGB images using the ResNet18 model with 100 × 100 patches.

Colors indicate prediction overlays:  and  = prediction,  = ground truth, ,  and  = overlap. Performance metrics shown per example: Acc, F1-score, AUC, and MCC.

## Usage Notes

HSI-AgriFoodAnomaly was designed as a reusable dataset for foreign-object detection in a specific agri-food inspection scenario based on an oat, white-chocolate, and dark-chocolate mixture. Although the dataset includes several foreign-object material families, the released pixel-level masks are binary and category-agnostic. Material information is available at scene level through the naming convention and dataset organisation, but the dataset should not be interpreted as a validated multi-class material-recognition benchmark.

The RGB renderings are provided for visual inspection and conventional image-based workflows. They were generated using SpectrononPro’s built-in spectral-to-RGB rendering function; therefore, they should not be interpreted as a physically calibrated simulation of a specific RGB camera spectral response function.

The baseline experiments are provided only as technical validation and usability checks. They should not be interpreted as a comprehensive benchmark of hyperspectral classification, segmentation, band selection, or real-time deployment methods. More advanced studies, including band selection, spectral dimensionality reduction, data augmentation, transformer-based models, segmentation benchmarks, multi-class material recognition, and real-time deployment profiling, represent possible future uses of the dataset.

Although the oat–chocolate matrix provides substantial intra-background variability in texture, packing density, colour, reflectance, and occlusion, the dataset remains limited to a single food background. Therefore, results obtained on HSI-AgriFoodAnomaly should not be assumed to generalise directly to other agri-food matrices without additional domain-shift evaluation.

## Data Availability

The dataset^28^ is publicly available at: https://doi.org/10.57745/QTLG7X.

## Code availability

The source code is publicly available at: https://github.com/lsllabisen/HSI-AgriFoodAnomaly-Dataset.git.
